# Small litter size impairs spatial memory and increases anxiety- like behavior in a strain-dependent manner in male mice

**DOI:** 10.1038/s41598-018-29595-0

**Published:** 2018-07-26

**Authors:** Ali-Akbar Salari, Hanieh Samadi, Judith R. Homberg, Morteza Kosari-Nasab

**Affiliations:** 1 Drug Applied Research Center, Tabriz University of Medical Sciences, Tabriz, Iran; 2 Salari Institute of Cognitive and Behavioral Disorders (SICBD), Alborz, Karaj, Iran; 3 Department of Cognitive Neuroscience, Centre for Neuroscience, Donders Institute for Brain, Cognition, and Behaviour, Radboud University Medical Centre, Nijmegen, The Netherlands

## Abstract

Early life overfeeding is associated with cognitive decline and anxiety-like behaviors in later life. It is not clear whether there are individual differences in the effects of early life overfeeding and what the underlying mechanistic pathways are. We investigated the long-lasting effects of small litter size, an experimental manipulation to induce neonatal overfeeding, in two strains of mice, C57BL/6 and NMRI. We measured body weight, learning and memory, anxiety-related behaviors, interleukin-(IL)-1β and brain-derived-neurotrophic-factor (BDNF) levels in the hippocampus, and both basal and stress corticosterone levels in adult mice which have been nursed in small litters compared with those from control litters. Our findings showed that small litter size led to increased body weight in both strains of mice. Small litter size significantly decreased spatial memory and hippocampal BDNF levels, and increased hippocampal IL-1β, in NMRI mice, but not C57BL/6 mice. Interestingly, we found that small litter size resulted in a significant increase in anxiety-like behaviors and stress-induced corticosterone in NMRI mice, whereas small litter size reduced anxiety-like symptoms and stress-induced corticosterone levels in C57BL/6 mice. These data show that small litter size, which is life-long associated with increased body weight, affects memory and anxiety-related behaviors in a strain-dependent manner in male mice. This suggests that there are individual differences in the developmental consequences of early life overfeeding.

## Introduction

The early postnatal period is a critical phase during which the systems that regulate metabolism and the brain undergo major developmental changes. Disturbances during this period can have long-lasting effects and lead to emotional impairments and cognitive decline in later life^1–3^. Regarding emotional impairments, several studies reported a significant positive association between overweight during childhood and psychological problems in later life such as anxiety^4–6^. Since anxiety is related to changes in the functioning of the hypothalamus-pituitary-adrenal (HPA)-axis^7,8^, it is plausible that early life changes in feeding alter the programming of the HPA-axis, to cause these problems in later life. In support, a relationship between early changes in feeding and HPA-axis functioning has been demonstrated in humans^9^. Regarding cognition, there is consistent evidence linking maternal obesity, which can influence neonatal nutrition, with lower cognitive function in children^10^, such as lower performance in pattern construction^11^, lower executive control functions^12,13^, and reduced action monitoring^14^. These cognitive impairments can have negative implications for daily life functioning and express a need for intervention. However, first further understanding is required; insight in underlying mechanisms and potential individual differences in the developmental consequences of neonatal overfeeding may help the design of personalized interventions.

Animal models are helpful to further understand how neonatal overfeeding affects later life emotion and cognition, as they allow precise control over environmental factors such the timing of nutritional changes as well as invasive brain studies. In rodents, neonatal overfeeding can be induced by reducing litter sizes from 16 to 4, which reduces the competition for food^15,16^. This approach leads to increased neonatal food intake, and thereby an overweight phenotype starting during the second postnatal week that was maintained into adulthood in both males and females^17^, and associated with a substantial increase in body fat^18^. Absolute juvenile and adult food intake is not altered and juvenile and adult small litter rats even eat less chow per gram body weight than the control litter rats in the active phase. Potentially, the life-long increased body weight relates to reduced whole body energy expenditure, as found in juvenile, but not adult small litter rats^18^. In adulthood, females from small litters showed enhanced exploratory behavior and reduced anxiety in the elevated plus maze^17^. Altered programming of the HPA-axis may play a role in these anxiety-related changes. For instance, neonatal overfed rats displayed increased activation of the paraventricular nucleus of the hypothalamus (PVN) in response to restraint stress^17^. The PVN is part of the HPA-axis, implying that the HPA-axis of neonatally overfeed animal is hyperreactive. In support, another study revealed that male rats raised in a small litter demonstrated accelerated HPA-axis maturation during development, accompanied by elevated stress-induced corticosterone secretion in adulthood^7^.

To the best of our knowledge, there are very few studies that investigated the effect of neonatal overfeeding on cognition. One study reported that neonatal overfeeding led to poor performance in radial arm maze and novel object recognition tests relative to controls^19^. However, the underlying mechanisms remain elusive. Proper functioning of memory processes, which are key in most types of cognitive functions, has been shown to highly depend on the integrity and correct functioning of the hippocampus. Western and high-fat-high sucrose diets in adulthood induce chronic low-grade inflammation in the hippocampus^20–23^. Injection of the inflammatory cytokine IL-1β in the rodent brain is sufficient to impair memory^24,25^. Besides inflammatory factors, also hippocampal brain-derived neurotrophic factor (BDNF) is affected by the consumption of diets high in fat and sugar^26,27^. Neurotrophic factors like BDNF play a crucial role in survival, maintenance and growth of neurons, and can regulate gene expression and long-term memory^28^. Potentially, low grade inflammation and changes in neurotrophic factor signaling play a role in the effects of neonatal overfeeding on cognition.

**Figure 1:**
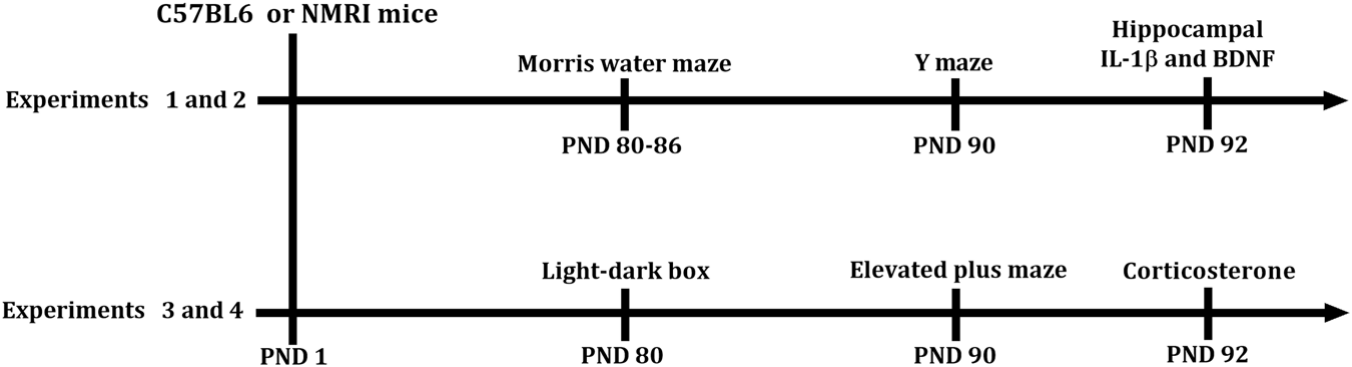
Experimental design: Experiments 1 and 2 investigate the effects of neonatal overfeeding in C57BL/6 and NMRI mice on spatial memory in the Morris water maze and Y maze tests, and IL-1β and BDNF levels in the hippocampus. Experiments 3 and 4 examine the effects of neonatal overfeeding in C57BL/6 and NMRI mice on anxiety-like behaviors in light-dark box and elevated plus maze tests, and basal and stress-induced serum corticosterone levels.

Here, we studied the effect of small litter size, an experimental manipulation that increases neonatal overfeeding, on emotion regulation and cognition in mice. We used mice varying in their innate level of anxiety, namely NMRI and C57BL/6 mice. We previously found that early life exposure to maternal infection increased anxiety in NMRI offspring, while it reduced anxiety in C57BL/6 offspring^29^. Strain differences thus have a great effect on behavioral outcomes, like trait anxiety in humans can be of great influence on emotion regulation and cognition^30^. In this study we manipulated the neonatal nutritional environment by culling half of the litters. Pups were tested as adults in tests for anxiety, with linkage to HPA-axis measurements, and tests for learning and memory, with linkage to hippocampal measurement of IL-1β and BDNF (The design of the experiments is illustrated in Fig. 1).

## Results

### Body weight

**Figure 2:**
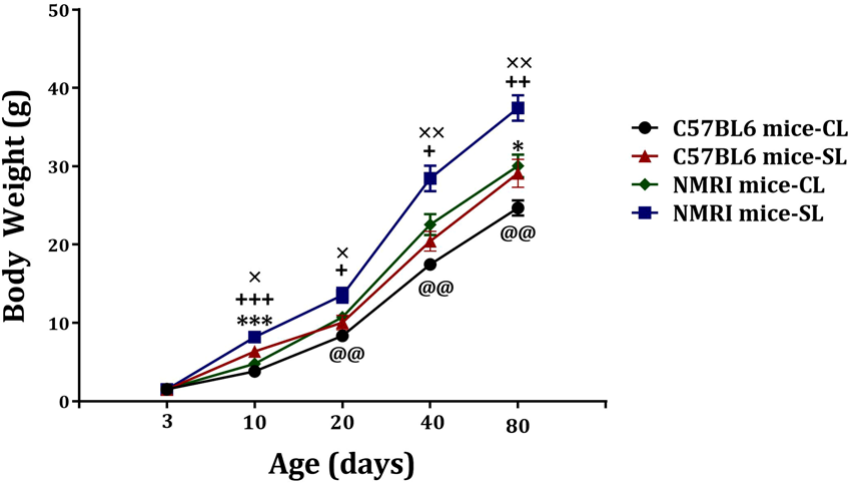
Effects of neonatal overfeeding on body weight in C57BL/6 and NMRI mice. Data are expressed as mean ± S.E.M. (N = 12). Significant differences following one-way ANOVA: ^*^P < 0.05 and ^***^P < 0.001, C57BL/6-SL mice compared to C57BL/6-CL mice; +*P* < 0.05, ++*P* < 0.01 and +++*P* < 0.001, NMRI-SL mice compared to NMRI-CL mice; @@*P* < 0.01, C57BL/6-CL mice compared to NMRI-CL mice; ×*P* < 0.05 and ××*P* < 0.01, C57BL/6-SL mice compared to NMRI-SL mice.

Small litter size is expected to increase body weight. Body weight was measured from P3 until P80. As illustrated in Fig. 2, a three-way repeated measure ANOVA revealed a main effect for day (f_(2.39,105.44)_ = 616.33, p < 0.001), strain (F_(1,44)_ = 62.48, p < 0.001) and litter size (F_(1,44)_ = 47.84, p < 0.001). While the day x strain x litter size interaction did not reach significance (F_(2.39,105.44)_ = 8.82, NS), significant day x strain (F_(2.39,105.44)_ = 10.14, p < 0.001) and day x litter size (F_(2.39,105.44)_ = 5.47, p = 0.003) interactions were observed. Further analyses revealed a significantly higher body weight in C57BL6 SL compared to C57BL6 CL mice at day 10 (F_(1,22)_ = 19.78, p < 0.001) and 80 (F_(1,22)_ = 4,663, p = 0.042), and in NMRI SL compared to NMRI CL mice across all days (F > 6.5, p < 0.05), except for day 3. The analysis also indicated significant differences between the NMRI and C57BL6 mouse strains in both CL (F > 8.9, p < 0.01) and SL (F > 6.5, p < 0.05) groups. In sum, small litter size is indeed associated with increased body weight, in both the NMRI and C57BL strains.

### Open Field, Locomotor Activity

As shown in Fig. S2, there were no significant interactions (F_(1,44)_ = 0.08, NS; F_(1,44)_ = 0.13, NS) and main effects of strain (F_(1,44)_ = 0.45, NS; F_(1,44)_ = 0.75, NS) and litter size (F_(1,44)_ = 1.59, NS; F_(1,44)_ = 1.48, NS) for both total line crossings and rearing, respectively, in the open field test. No significant change was found in the locomotor activity of mice.

### Morris Water Maze test

**Figure 3:**
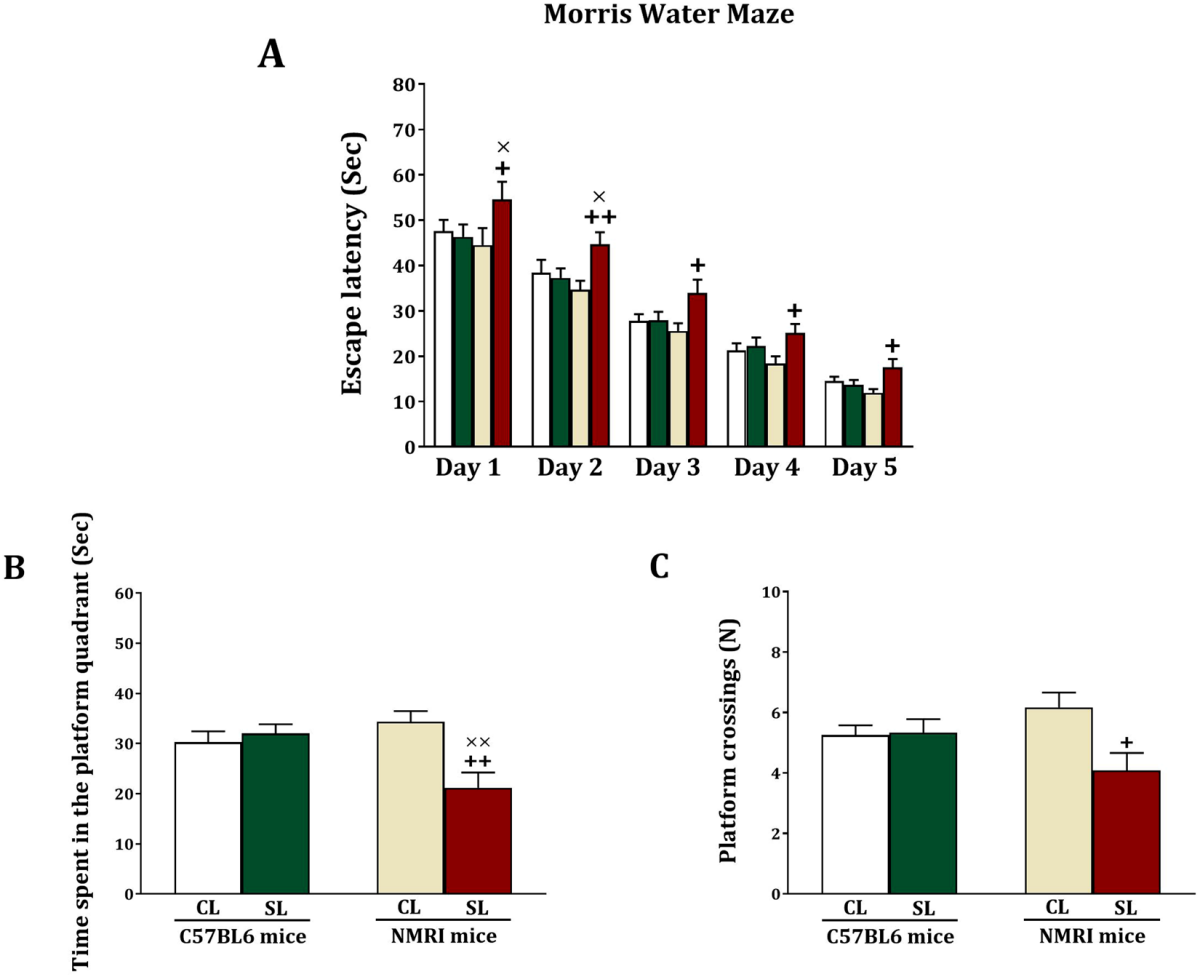
Effects of neonatal overfeeding in C57BL/6 and NMRI mice on learning and memory in the Morris water maze test. Values are presented as mean + S.E.M. (N = 12) of escape latency (**A**), time spent in the platform quadrant (**B**) or platform crossing (**C**). Significant differences following one-way ANOVA: +*P* < 0.05 and ++*P* < 0.01, compared to NMRI-CL mice; ×*P* < 0.05 and ××*P* < 0.01, compared to C57BL/6-SL mice.

To assess whether small litter size affects cognition, we measured spatial memory in the Morris Water Maze test. For the acquisition phase of this test, repeated measures ANOVA did not reveal significant day x strain (F_(2.93,129.11)_ = 0.25, NS), day x litter size (F_(2.93,129.11)_ = 0.18, NS), and day x strain x litter size (F_(2.93,129.11)_ = 0.39, NS) interactions (Fig. 3A). However, a between-subject analysis elucidated that there are significant litter size (F_(1,44)_ = 16.02, p < 0.001), and strain x litter size (F_(1,44)_ = 19.97, p < 0.001) interaction, although there was no significant strain effect (F_(1,44)_ = 2.15, NS). Subsequent analyses revealed that there were no litter size differences for C57BL6 mice (F > 0.35, NS), while NMRI SL mice took significantly longer to find the platform across all days of testing (F > 6.3, p < 0.05). Significant differences were also observed between NMRI SL and C57BL6 SL groups at days 1 (F_(1,22)_ = 5.21, p = 0.032) and 2 (F_(1,22)_ = 5.05, p = 0.035), in such a way that the NMRI SL mice needed more time to find the platform. Since there was a learning curve across the 4 trials of the first day of acquisition (Fig. S1), these findings point to impaired learning in NMRI SL mice.

For the spatial memory test, when the mice were expected to swim to the quadrant where the platform was previously located, we obtained significant litter size (F_(1,44)_ = 5.98, p = 0.019) and stain x litter size interactions (F_(1,44)_ = 10.21, p = 0.003) (Fig. 3B). There was, however, no significant strain effect (F_(1,44)_ = 2.09, NS). Further analyses revealed that there were no litter size differences among the C57BL6 mice (F_(1,22)_ = 0.38, NS), whereas NMRI SL mice, compared to NMRI CL mice, spent significantly less time in the quadrant where the platform was located during acquisition (F_(1,22)_ = 12.62, p = 0.002). In addition, NMRI SL mice exhibited a significant decrease in the time spent in the platform quadrant (F_(1,22)_ = 9.34, p = 0.006) as compared to C57BL6 SL mice. Regarding the number of platform crossings (Fig. 3C), we found a significant litter size effect (F_(1,44)_ = 4.49, p = 0.04) and strain x litter size (F_(1,44)_ = 5.27, p = 0.026) interaction. There was no significant strain effect (F_(1,44)_ = 0.125, NS). Further testing revealed that there were no litter size differences for the C57BL6 group (F_(1,22)_ = 0.22, NS), while the number of platform crossings were significantly lower for the NMRI SL group compared to the NMRI CL group (F_(1,22)_ = 7.481, p = 0.012). No significant differences were found between NMRI SL and C57BL6 SL mice for the platform crossings. In sum, small litter size affects cognition in NMRI mice, but not C57BL mice.

### Y Maze test

**Figure 4:**
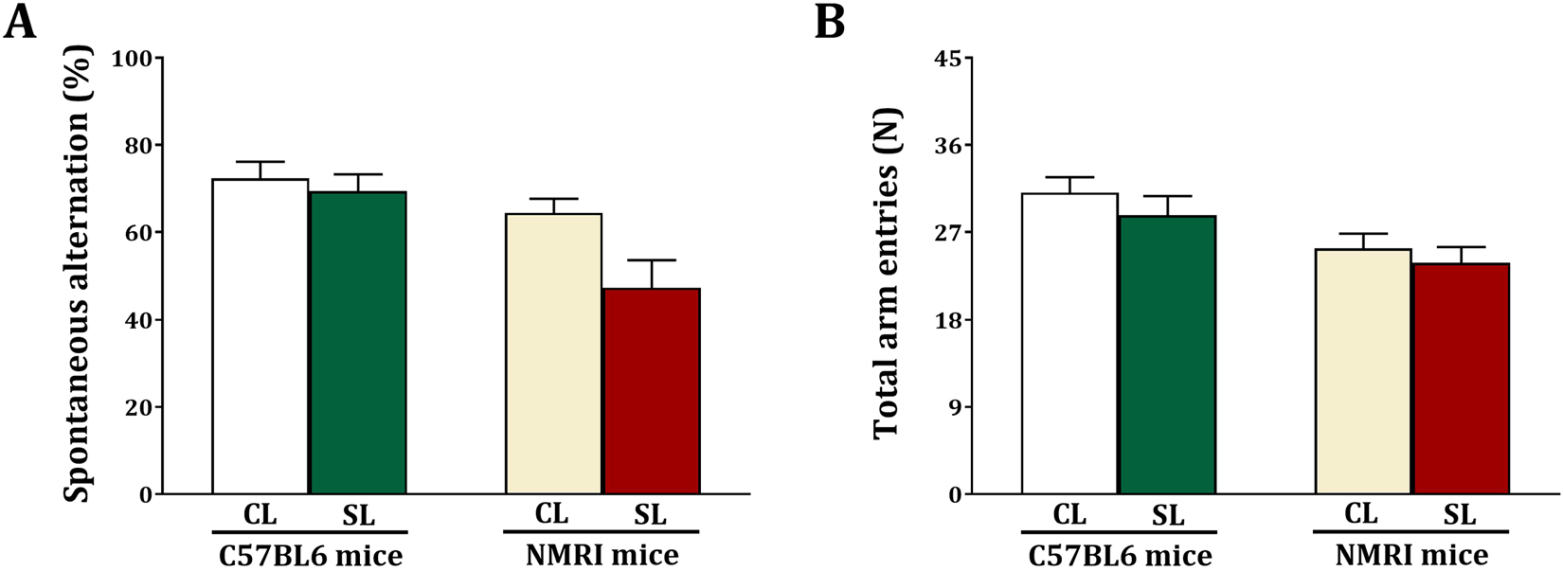
Effects of neonatal overfeeding in C57BL/6 and NMRI mice on memory in the Y maze test. Values are presented as mean + S.E.M. (N = 12) of spontaneous alternation (**A**) and total arm entries (**B**).

The effect of small litter size on cognition was also investigated using the Y-maze test, measuring spontaneous alternation (Fig. 4A). A two-way analysis revealed a main effect for strain (F_(1,44)_ = 11.27, p = 0.002) and litter size (F_(1,44)_ = 5.09, p = 0.029). However, there was no significant interaction between strain x litter size (F_(1,44)_ = 2.51, NS). The number of entries are shown in Fig. 4B. Statistical analysis revealed a significant strain effect (F_(1,44)_ = 10.05, p = 0.003), without a litter size (F_(1,44)_ = 1.29, NS) and strain x litter size (F_(1,44)_ = 0.61, NS) effect. These data show that strain and small litter size affect spontaneous alternation in mice.

### IL-1β and BDNF protein

**Figure 5:**
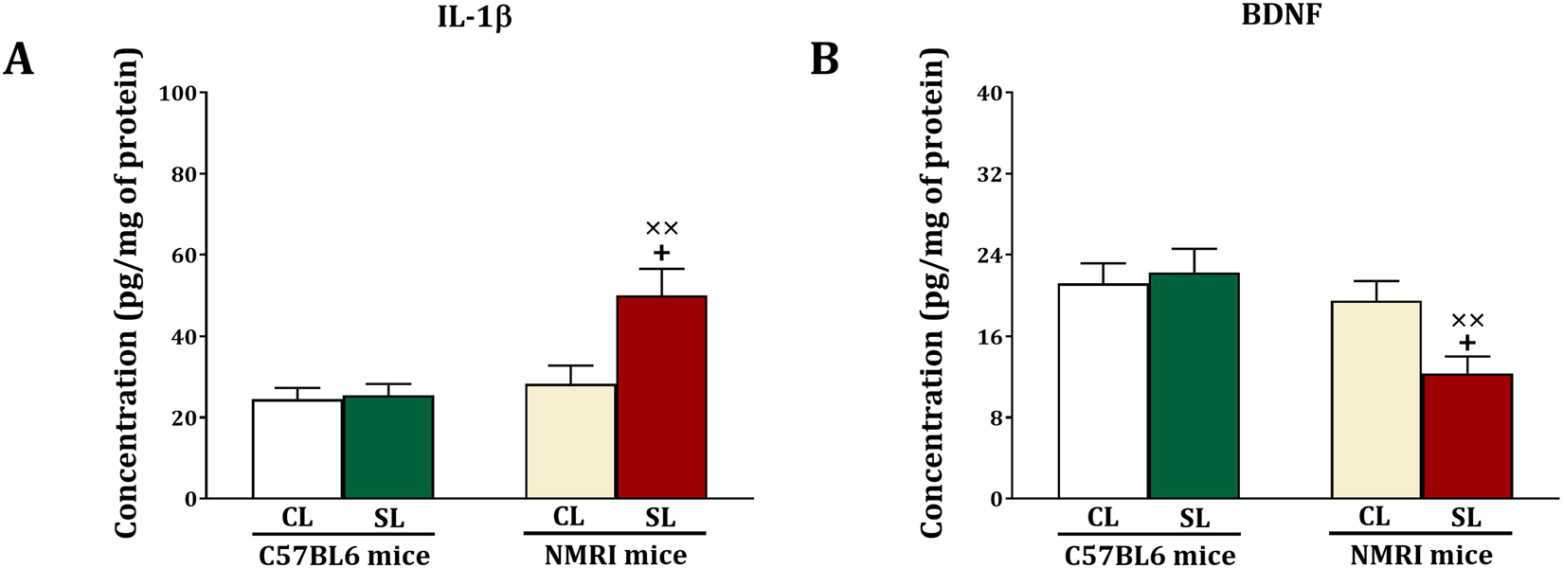
Effects of neonatal overfeeding in C57BL/6 and NMRI mice on IL-1β (**A**) and BDNF (**B**) levels in the hippocampus. Values are presented as mean + S.E.M. (N = 12) concentration of BDNF and IL-1β. Significant differences following one-way ANOVA: +*P* < 0.05, compared to NMRI-CL mice; ××*P* < 0.01, compared to C57BL/6-SL mice.

As illustrated in Fig. 5A,B, following Morris Water Maze and Y Maze testing we sacrificed the animals to measure hippocampal IL-1β and BDNF proteins using ELISA. We obtained significant strain (F_(1,44)_ = 10.32, p = 0.002), litter size (F_(1,44)_ = 6.61, p = 0.014) and strain x litter size (F_(1,44)_ = 5.59, p = 0.022) interactions for IL-1β. Further testing revealed that IL-1β levels are not different between C57BL6 SL and CL mice (F_(1,22)_ = 0.53, NS), but are increased in NMRI SL versus CL mice (F_(1,22)_ = 7.63, p = 0.011). Statistical analysis also showed a significant increase in IL-1β levels (F_(1,22)_ = 12.21, p = 0.002) in NMRI SL mice relative to C57BL6 SL mice.

Regarding BDNF, there were significant strain (F_(1,44)_ = 8.44, p = 0.006) and strain x litter size (F_(1,44)_ = 4.28, p = 0.044) effects, but there was no litter size (F_(1,44)_ = 2.32, NS) effect. It was found that the strain and strain x litter size effects were attributed to a decrease in BDNF levels in NMRI SL versus NMRI CL mice (F_(1,22)_ = 7.92, p = 0.01). There were no BDNF differences between C57BL6 SL and CL mice (F_(1,22)_ = 0.12, NS). A significant decrease also existed in BDNF levels (F_(1,22)_ = 11.85, p = 0.002) in NMRI SL mice compared to C57BL6 SL mice. Thus, small litter size induced cognitive impairment in NMRI mice is associated with higher levels of IL-1β and lower levels of BDNF in the hippocampus.

### Light-Dark Box

**Figure 6:**
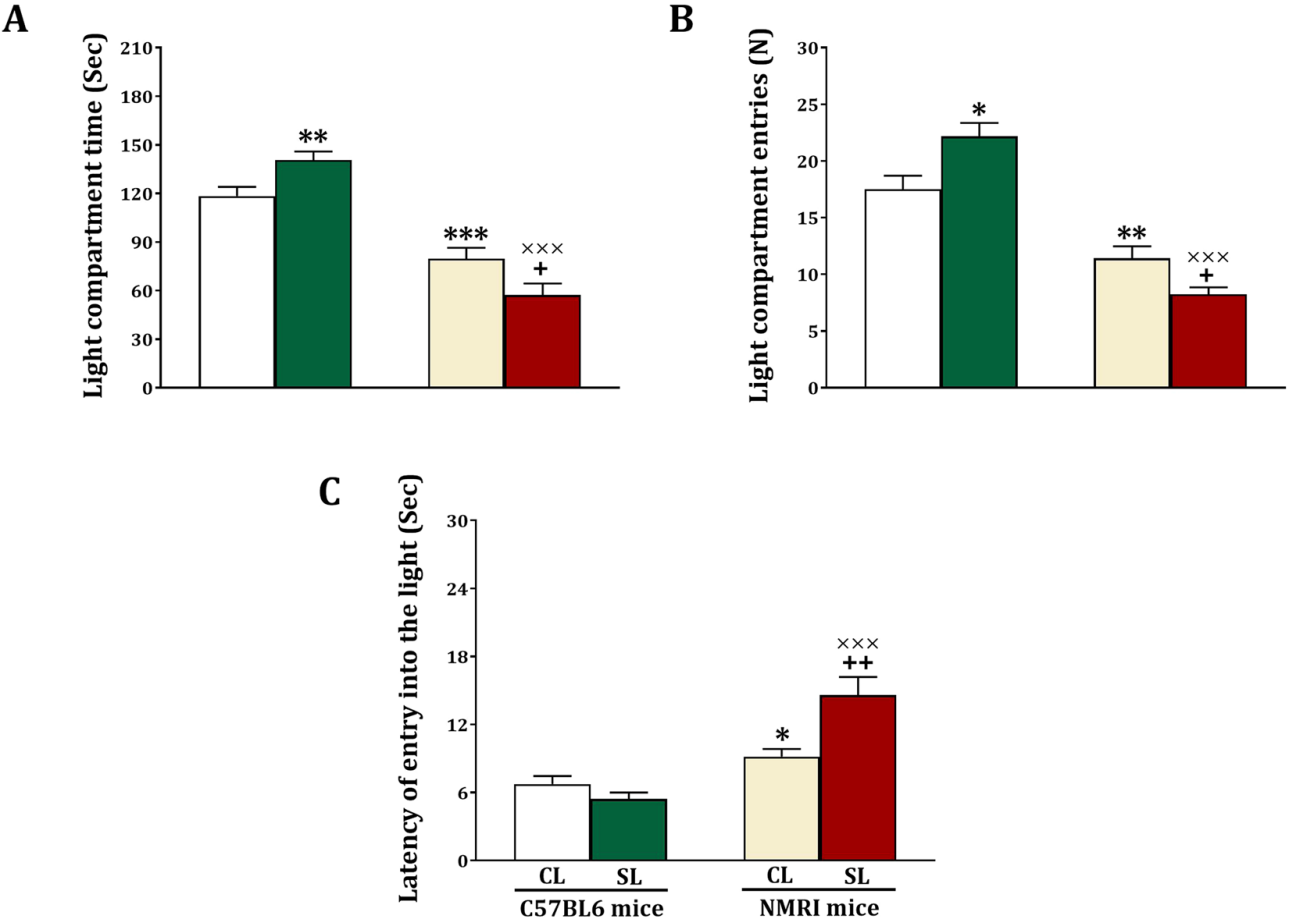
Effects of neonatal overfeeding in C57BL/6 and NMRI mice on anxiety-like behaviors in light-dark box. Values are presented as mean + S.E.M.(N = 12) of light compartment time (**A**), light compartment entries (**B**) or latency of entry into the light (**C**). Significant differences following one-way ANOVA: ^*^P < 0.05, ^**^P < 0.01 and ^***^P < 0.001, compared to C57BL/6-CL mice; +*P* < 0.05 and ++*P* < 0.01, compared to NMRI-CL mice; ×××*P* < 0.001, compared to C57BL/6-SL mice.

Besides cognition, small litter size can also affect anxiety. To test this, anxiety was measured in the Light-Dark box. We measured the time the animals spent in the light compartment (Fig. 6A) and found significant strain (F_(1,44)_ = 95.63, p < 0.001) and strain x litter size (F_(1,44)_ = 12.75, p < 0.001) effects, but no litter size (F_(1,44)_ = 0.98, NS) effect. Further testing revealed that the time spent in the light compartment is increased in C57BL6 SL versus CL mice (F_(1,22)_ = 8.17, p = 0.009), and decreased in NMRI SL versus CL mice (F_(1,22)_ = 5.24, p = 0.032). Furthermore, among the CL mice those with an NMRI background compared to mice with a C57BL6 background spent less time in the light compartment (F_(1,22)_ = 19.33, p < 0.001). Likewise, among the SL mice those with an NMRI background compared to mice with a C57BL6 background spent less time in the light compartment (F_(1,22)_ = 88.81, p < 0.001).

Regarding light compartment entries (Fig. 6B), we obtained significant strain (F_(1,44)_ = 92.65, p < 0.001) and strain x litter size (F_(1,44)_ = 14.21, p < 0.001) effects, but no litter size (F_(1,44)_ = 0.52, NS) effect. Posthoc testing revealed that the number of light compartment entries is significantly increased in SL versus CL C57BL6 mice (F_(1,22)_ = 7.71, p = 0.011), and significantly decreased in SL versus CL NMRI mice (F_(1,22)_ = 6.71, p = 0.017). Postdoc testing also revealed that among the CL mice, those with an NMRI background compared to mice with a C57BL6 background entered less in the light compartment (F_(1,22)_ = 14.54, p < 0.001). Among the SL mice those with an NMRI background compared to mice with a C57BL6 background entered less in the light compartment (F_(1,22)_ = 109.21, p = 0.001).

Finally, for the latency to entry the light compartment there were significant strain (F_(1,44)_ = 34.07, p = 0.001), litter size (F_(1,44)_ = 4.23, p = 0.046) and strain x litter size (F_(1,44)_ = 11.57, p < 0.001) effects (Fig. 6C). Further testing revealed that there were no litter size differences for the C57BL6 mice (F_(1,22)_ = 2.11, NS), but that the latency was higher in NMRI SL versus NMRI CL mice (F_(1,22)_ = 9.47, p = 0.005). Furthermore, among the CL (F_(1,22)_ = 6.02, p = 0.022) and SL (F_(1,22)_ = 28.30, p = 0.001) mice, NMRI mice showed a higher latency compared to C57BL6. In sum, small litter size decreased anxiety in C57BL6 mice, and increased anxiety in NMRI mice.

### Elevated plus maze

**Figure 7:**
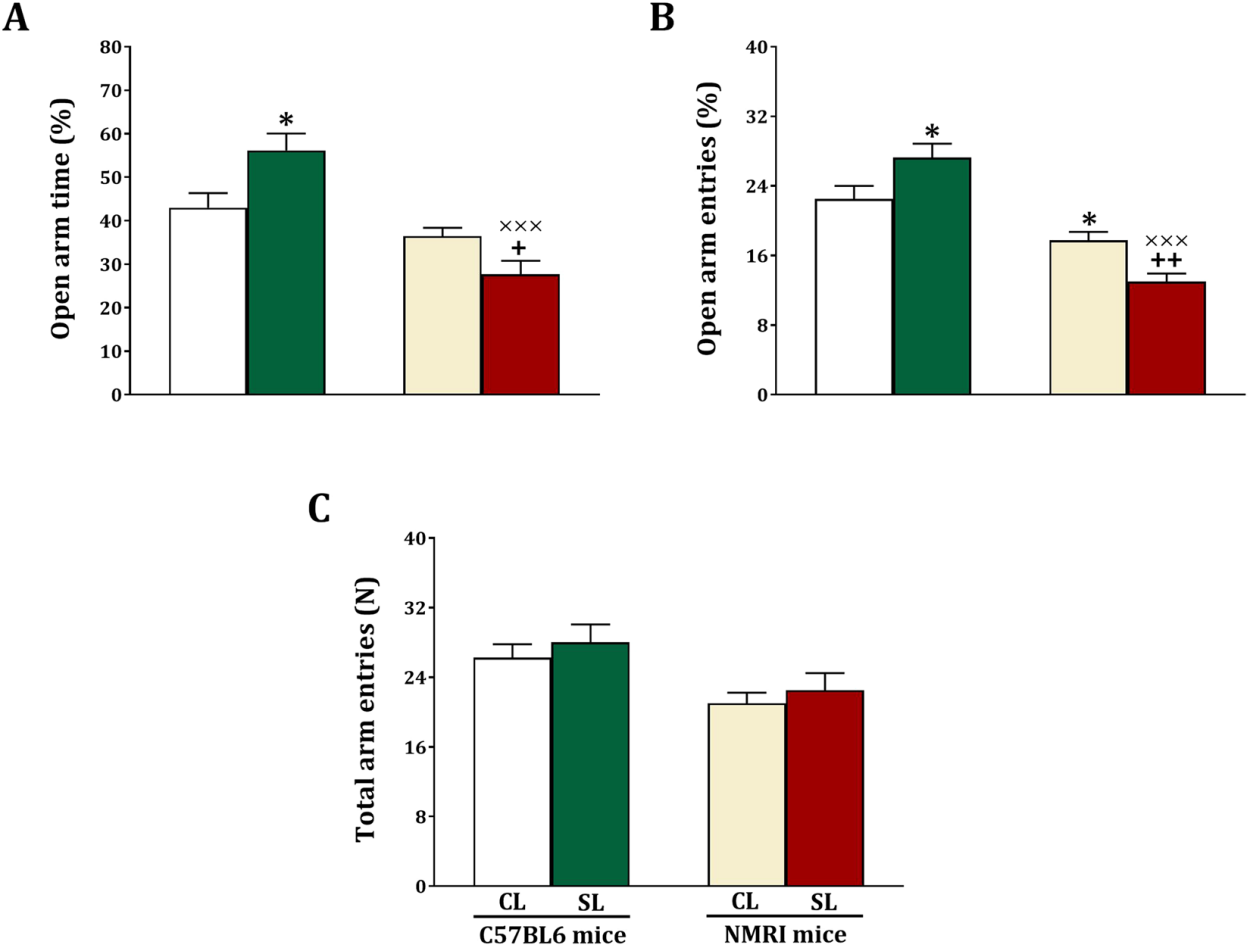
Effects of neonatal overfeeding in C57BL/6 and NMRI mice on anxiety-like behaviors in the elevated plus maze test. Values are presented as mean + S.E.M. (N = 12) of open arm time % (**A**), open arm entries % (**B**) or locomotor activity (**C**). Significant differences following one-way ANOVA: ^*^P < 0.05, compared to C57BL/6-CL mice; +*P* < 0.05 and ++*P* < 0.01, compared to NMRI-CL mice; ×××*P* < 0.001, compared to C57BL/6-SL mice.

The effect of small litter size on anxiety was also assessed using the elevated plus maze test. In this test we first measured open arm time and obtained significant strain (F_(1,44)_ = 31.10, p < 0.001) and strain x litter size (F_(1,44)_ = 12.13, p < 0.001) effects, but no litter size (F_(1,44)_ = 0.49, NS) effects (Fig. 7A). The analyses also showed that open arm time is higher for C57BL6 SL compared to C57BL6 CL mice (F_(1,22)_ = 6.62, p = 0.017), and lower for NMRI SL compared to NMRI CL mice (F_(1,22)_ = 5.71, p = 0.026). Furthermore, open arm time was lower in NMRI SL mice compared to C57BL6 SL mice (F_(1,22)_ = 33.29, p < 0.001), but not among C57BL6 and NMRI CL mice (F_(1,22)_ = 2.85, NS).

Regarding open arm entries (Fig. 7B), we obtained significant strain (F_(1,44)_ = 54.67, p < 0.001) and strain x litter size (F_(1,44)_ = 13.670, p < 0.001) effects. There was not a significant litter size effect (F_(1,44)_ = 1, NS). Subsequent testing revealed that open arm entries higher for C57BL6 SL compared to C57BL6 CL mice (F_(1,22)_ = 4.67, p = 0.042), and lower for NMRI SL compared to NMRI CL mice (F_(1,22)_ = 12.71, p = 0.002). Furthermore, open arm entries lower in NMRI SL mice compared to C57BL6 SL mice (F_(1,22)_ = 59.53, p < 0.001), and in NMRI CL compared to C57BL6 CL mice (F_(1,22)_ = 7.07, p = 0.014).

In Fig. 7C we illustrate locomotor activity in the elevated plus maze test. We obtained a significant strain effect (F_(1,44)_ = 9.57, p = 0.003), but the litter size (F_(1,44)_ = 0.87, NS) and strain x litter size (F_(1,44)_ = 0.94, NS) effects were not significant. Furthermore, locomotor activity (F_(1,22)_ = 3.7, p = 0.014) lower in NMRI CL mice compared to C57BL6 CL mice. These findings together show that also in the elevated plus maze test small litter size decreased anxiety in C57BL6 mice, and increased anxiety in NMRI mice. These effects were independent of activity.

### Baseline and stress-induced serum corticosterone levels

**Figure 8:**
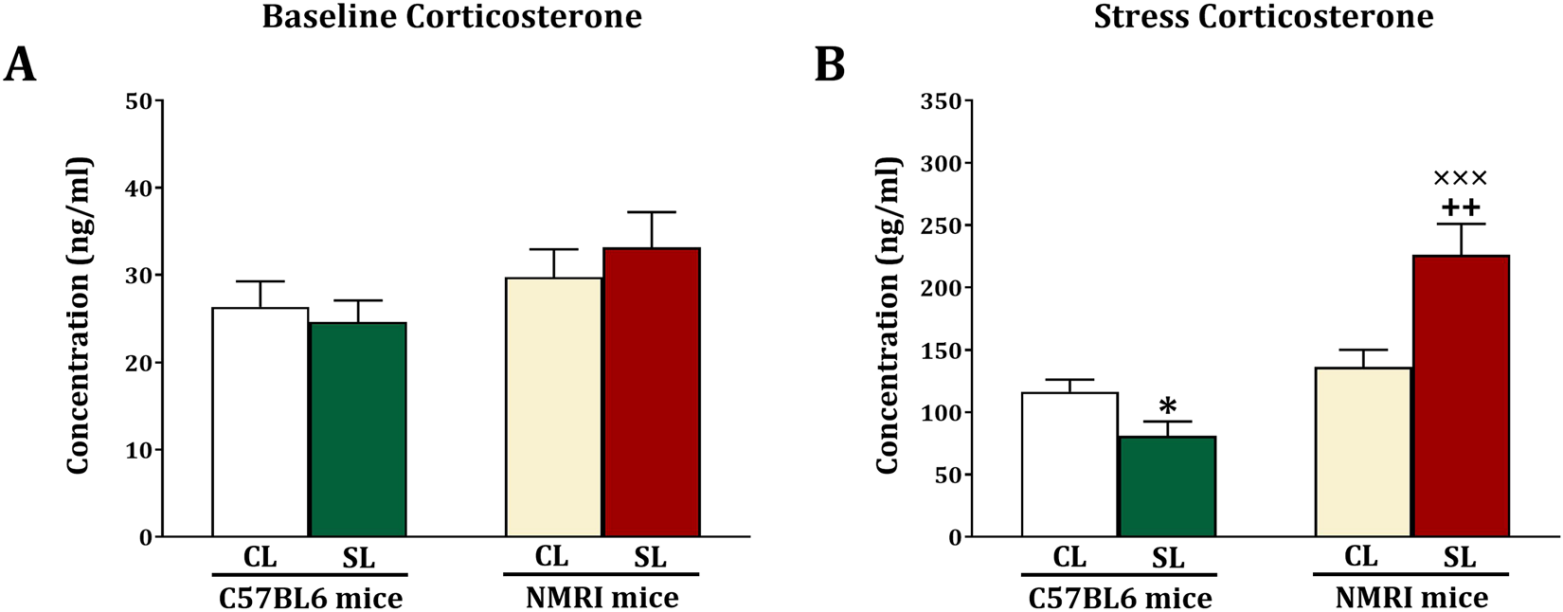
Effects of neonatal overfeeding in C57BL/6 and NMRI mice on basal (**A**) and stress-induced (**B**) corticosterone levels. Values are presented as mean + S.E.M. (N = 12) serum corticosterone levels. Significant differences following one-way ANOVA: ^*^P < 0.05, compared to C57BL/6-CL mice; ++*P* < 0.01, compared to NMRI-CL mice; ×××*P* < 0.001, compared to C57BL/6-SL mice.

In mice that were sacrificed 2 days after elevated plus maze testing we measured baseline serum corticosterone levels. As shown in Fig. 8A, there were no significant strain (F_(1,44)_ = 3.49, NS), litter size (F_(1,44)_ = 0.07, NS) and strain x litter size (F_(1,44)_ = 0.63, NS) effects. In mice that were 20 min after elevated plus maze testing we measured stress-induced serum corticosterone levels (Fig. 8B). We obtained significant strain (F_(1,44)_ = 26.14, p < 0.001) and strain x litter size (F_(1,44)_ = 15.14, p < 0.001) effects, but the litter size effect was not significant (F_(1,44)_ = 2.84, NS). Further testing revealed that among the C57BL6 mice, the SL group exhibited lower stress-induced serum corticosterone levels compared to the CL group (F_(1,22)_ = 5.54, p = 0.028). Additionally, among the NMRI mice, those of the SL group had higher serum corticosterone levels compared to the ones in the CL group (F_(1,22)_ = 9.96, p = 0.005). Finally, there were no strain differences among the CL mice (F_(1,22)_ = 1.34, NS), but among the SL mice, the NMRI mice had higher serum corticosterone levels compared to C57BL6 mice (F_(1,22)_ = 28.03, p < 0.001). These data show that increased anxiety induced by small litter size in NMRI mice is associated with increased stress-induced serum corticosterone levels. Furthermore, in line with their decreased anxiety, C57BL6 SL mice showed a decreased stress-induced serum corticosterone response.

### Correlations between Morris Water Maze and Y Maze performance, and IL-β and BDNF levels

Correlation analyses revealed that there was a significant positive correlation between time spent in the platform and spontaneous alternation in NMRI CL mice (r = 0.732, p = 0.007) and NMRI SL mice (r = 0 0.874, p < 0.001) (Fig. S3A, Supplementary information). We also observed that time spent in the platform quadrant correlated with BDNF levels in NMRI CL mice (r = 0.739, p = 0.006) and NMRI SL mice (r = 0.870, p < 0.001) (Fig. S3B, Supplementary information). Furthermore, we found significant correlations between spontaneous alternation in the Y Maze and hippocampal IL-β levels in NMRI CL mice (r = −0.887, p < 0.001) and NMRI SL mice (r = −0.945, p < 0.001) (Fig. S3C, Supplementary information). Finally, IL-β and BDNF protein levels were negatively correlated in NMRI CL (r = −0.719, p = 0.009) and NMRI SL mice (r = −0.816, p = 0.001) (Fig. S3D, Supplementary information).

### Correlations between light-dark box and elevated plus maze exploration, and stress-induced plasma corticosterone levels

When correlating the light-dark box, elevated plus maze and serum corticosterone levels, we found a significant positive correlation between the time spent in the light compartment and the open arm time in C57BL6 CL (r = 0.768, p = 0.004), C57BL6 SL (r = 0.803, p = 0.002), NMRI CL (r = 0.781, p = 0.003) and NMRI SL (r = 0.787, p = 0.002) mice (Fig. S4A,B, Supplementary information). Furthermore, open arm time in the elevated plus maze correlated positively with stress-induced corticosterone levels in C57BL6 CL (r = −0.848, p < 0.001), C57BL6 SL (r = −0.862, p < 0.001), NMRI CL (r = −0.766, p = 0.004) and NMRI SL (r = −0.954, p < 0.001) mice (Fig. S2C,D, Supplementary information).

## Discussion

We studied the effect of small litter size on cognition (learning and memory) and anxiety, using two tests for each of these outcomes. We found that small litter size decreased the acquisition and expression of spatial memory in the Morris Water Maze task and decreased spontaneous alternation in the Y-maze, in the NMRI but not C57BL6 mice. Both tests are hippocampus dependent^31,32^. We also found that both IL-1β and BDNF levels were reduced in the hippocampus of NMRI but not C57BL6 mice. Regarding anxiety-related behavior we noted increased anxiety in NMRI mice exposed to the small litter manipulation in the light-dark box and the elevated plus maze test. Yet, decreased anxiety was found for the small litter C57BL6 mice in the light-dark box and elevated plus maze test. Basal plasma corticosterone levels were not altered in the two lines of mice, but stress-induced plasma corticosterone levels were increased in small litter NMRI mice and decreased in small litter C57BL6 mice. Importantly, changes in anxiety were not related to changes in locomotor activity.

Our data show that the effects of small litter size are strongly strain dependent. In line with our previous findings^29^, opposite effects of an early life insult were found in NMRI compared to C57BL6 mice. Thus, while small litter size led to impaired cognition and increased anxiety in NMRI mice, decreased anxiety was found in C57BL6 mice. The NMRI mouse strain is outbred and the C57BL6 mouse strain is inbred, and they differ significantly in their baseline level of anxiety. Because C57BL6 mice are more reactive to threat compared to NMRI mice^33–35^, it may be that the range to observe an increase in anxiety was larger in NMRI mice compared to C57BL6 mice. Why the anxious C57BL6 mice became less anxious after rearing in small litters is unclear. The mouse strains are differentially sensitive to environmental stimuli^34^. Potentially, small litter size, leading to increased body weight, reduced sensitivity to environmental information and thereby reduced anxiety in C57BL6 mice. A previous study suggested that C57BL6 and NMRI mice also differ in hippocampal function, which may explain the cognitive differences between the two strains^36^, but evidence is too sparse for further speculation. Regardless of the explanation of the differences between the two mouse strains, we report here for the first time that small litter size, which leads to increased body weight, presumably due to neonatal overfeeding, is a trigger for the manifestation of C57BL6 and NMRI mouse strain differences at the level of anxiety and cognition.

The present finding that small litter size impaired cognition in NMRI mice corresponds to the finding that 3 weeks of overfeeding prior to weaning impaired cognition as measured in the radial maze (measuring working memory) and object recognition test (measuring object recognition memory)^19^. Furthermore, another study reported that female, but not male, rats subjected to overfeeding during the neonatal period displayed decreased anxiety, no changes in stress-induced corticosterone release, but increased stress-induced activity of the PVN, the hypothalamic part of the HPA-axis^17^. The decreased anxiety seems to parallel the decreased anxiety we found in small litter C57BL6 mice, although these were males and displayed decreased stress-induced serum corticosterone levels. Other studies reported that neonatal overfeeding is associated with increased basal and stress-induced corticosterone levels in rats, but sex was not reported and anxiety was not measured^7,37^. Since rats were used in these studies, species differences may explain why findings do not fully copy. Nonetheless, when taken data all together there is strong evidence that changes in the neonatal nutritional environment affects the cognitive and emotional domains of behavior.

We observed a significant positive correlation between anxiety on the elevated plus maze test and stress-induced corticosterone levels. Since corticosterone is the end product of the HPA-axis, this finding suggests that anxiety is related to HPA-axis function. Neonatal overfeeding has been shown to increase HPA-axis responsivity to stress. It was found that neonatally overfed rats exhibit microgliosis in the PVN, and increased hypothalamic expression of inflammatory factors^38^. While we did not measure inflammatory factors in the hypothalamus, given that hypothalamic inflammation is associated with HPA-axis activation^39^, it is plausible that such a mechanism contributes to the HPA-axis hyperactivity and increased anxiety as we observed in NMRI mice. Adrenal hypertrophy or changes in the expression of enzymes responsible for glucocorticoid metabolism, as reported for neonatally overfed rats^7,37^, could also be implicated in the present NMRI mouse findings.

We found that IL1β levels were increased, and that BDNF levels were decreased in the hippocampus of SL NMRI mice, which also displayed reduced memory function in the Morris Water Maze and Y-maze tasks. Furthermore, we found a negative correlation between IL1β and memory in the Y-maze task, and a positive correlation between BDNF levels and spatial memory in the Morris Water Maze task. These findings suggest that small litter size and associated increase in body weight leads to an immune response in the hippocampus, thereby impairing hippocampal function. In support, it has been reported, albeit in ageing rats, that a high fat diet leads to microglia activation and an increase in IL-1β levels, and thereby cognitive impairment^40^. BDNF positively regulates synaptic plasticity and cell proliferation in the hippocampus as well as spatial memory as measured in the Morris Water Maze test^31,41,42^. Accordingly, the reduced BDNF levels, which we found in the hippocampus of small litter NMRI mice and correlated with hippocampus dependent spatial memory in the Morris Water Maze task, may relate to reduced spatial memory through a decrease in neuroplasticity and neurogenesis.

This study is characterized by strengths as well as limiting factors. A major strength of the present study is that cognition and anxiety were assessed in two behavioral tests each. Since directional changes were similar for all tests per mouse strain, we can be confident that our findings are robust. A second strength is that we tested two mouse strains, which vary in their response to small litter size. This underlines how critical investigation of individual differences is. Also, some limitations have to be noted. First, only male mice were tested. We can therefore not generalize our findings to small litter size effects across male and female subjects. Body weight changes due to small litter size could influence task performance. For instance, heavier animals may move slower. This could have affected, for instance, Morris Water Maze performance. However, we obtained similar findings across two different tests for cognition and anxiety, and in the elevated plus maze and locomotor tests the null hypothesis for differences in locomotor activity between control and small litter size animals could not be rejected, rendering this possibility unlikely. Finally, we did not measure maternal care behavior, and therefore we cannot exclude the possibility that differences in maternal care have contributed to the observed small litter size effects. Yet, all pups were placed with another mother, and thus all mothers were treated in a comparable manner. Furthermore, it has previously been shown that litter sizes between 5–18 do not alter maternal care behavior^43^.

In summary, in this study we have demonstrated that small litter size, leading to increased body weight, can play a crucial role in the programming of adult cognition and anxiety-related behavior, at least in part through changes in hippocampal and HPA-axis function, respectively. Furthermore, we demonstrate individual differences in the effects of small litter size, and thereby putative effects of neonatal overfeeding. This indicates that neonatal overfeeding does not always have detrimental consequences, but rather interacts with individual differences in susceptibility to early life changes in the (nutritional) environment. This asks for investigation of susceptibility factors in humans in order to be able to target early interventions to specifically those that are expected to develop disadvantageously when exposure to neonatal overfeeding.

## Material and Methods

### Animals and Ethics

Fifty-five and forty-three timed pregnant C57BL6 and NMRI mice, respectively, were obtained from the animal house of “Salari Institute of Cognitive and Behavioral Disorders (SICBD)”. Animals were housed in a room with a 12:12 h light/dark cycle (lights on 08:00 AM) and temperature (23 ± 1 °C) with access to food and water *ad libitum*. These conditions were kept as a standard housing condition in all stages of the experiments^44^. All experimental procedures were approved by the Ethics Committee of SICBD (GN-94-01) and were performed in accordance with relevant guidelines and regulations.

### Litter manipulation

All pregnant animals were allowed to have normal delivery. The first day of birth was considered as postnatal day (PD) 0^45^. Based on previous studies, the animals were divided into two litter groups, control and small^38,46–48^. At PD1, litters were culled to 10 male pups in the control litter group (CL), and to 4 male pups in the small litter group (SL). Because this procedure may differentially affect litters with can differ in size and sex composition, the pups were removed from their mothers and randomly reallocated to new dams. No dam received any of her own pups, as previously described by Spencer’s team^17–19,38,48–51^. All litters were weaned at PD 28^52^ and housed 2 mice per cage until PD 80, when animals were subjected to behavioral tests. Only one male from each litter was randomly assigned for each group per experiment (N = 12). The body weight of offspring was recorded at PD 3, 10, 20, 40 and 80, to capture important phases in development.

### Behavioral tests

The experienced observers and recorded all parameters for each of the behavioral tests which were blind to the treatment. All behavioral tests were performed in a quiet room during the light period (between 12:00-16:00 h)^53^ under illumination of 75 lux. Animals were kept in the room for at least 1 h before the assessment. The design of the experiments is illustrated in Fig. 1. Animals in experiments 1 and 2 were subjected to the Morris water maze and Y-maze tests to measure spatial memory, and used to measure IL-1β and BDNF levels in the hippocampus. Animals in experiments 3 and 4 were subjected to the light-dark box and elevated plus maze tests to measure anxiety, and used to measure baseline and stress-induced plasma corticosterone levels.

#### Locomotor activity

To measure the locomotion in mice, the open field test was used as described previously by our group^53^. The apparatus consisted of a white wooden box (40 cm × 40 cm × 20 cm) with 16 squares (10 cm × 10 cm). The animal was gently placed in the center of the apparatus and allowed to move freely for 5 min. Two behavioral parameters, total line crossings and rears, were recorded as horizontal and vertical activities. A line crossing was considered when all four paws of the animal completely crossing a line and a rear was counted when the animal stood on its hind legs.

#### Morris water maze

The Morris water maze was performed as previously described^54^. The apparatus consisted of a black circular tank (100 cm diameter, 50 cm high), filled with water (23 ± 1 °C) and divided into four equally spaced quadrants (I, II, III, IV). A small circular escape platform (10 cm diameter, 24 cm high) was placed in the middle of one quadrant (III), 1 cm below the surface of the water. The task was conducted four times (4 trials; 90 s per trial, there was a 60-min intertrial interval) a day for 5 consecutive days. A trial was initiated by placing each animal into the tank at one of three starting positions (the sequence of the positions was selected randomly) facing the pool wall and allowing it to circumnavigate the pool in search of the escape platform. When the animal found the platform, it was allowed to remain on it for 20 s. If the animals did not find the platform at the end of the 90-s trial period, they were gently guided to it. Escape latency (second) was recorded to indicate the learning results. On day 6, probe trial (90 s) was performed after removing the escape platform from the pool. The time spent in the platform quadrant and the number of crossing the platform were measured to indicate the memory results.

#### Y maze

To evaluate spatial memory, spontaneous alternation behavior was measured in the Y maze as described previously^55^. Briefly, the Y maze was made of medium-density fibreboard in gray. The apparatus had three identical arms (labeled A, B and C) with equal angles (120°) between all arms. Each arm of Y maze was 40 cm long, 15 cm high and 8 cm wide. The apparatus was set up on the floor of the experimental room. During the 8-min test period, each mouse was placed at the end of one arm and allowed to explore the maze freely. The sequence and the number of entries into each arm were recorded manually. An arm entry was considered when the rats completely placed their four paws within the arm. A spontaneous alternation was considered as an actual alternation when a rat entered three different arms (i.e., ABC, CAB, or BCA but not BAB) on overlapping triplet sets. The percentage of spontaneous alternation was calculated as [(actual alternations/total number of arm entries − 2) × 100]. Total numbers of arm entries served as indicators of memory and locomotor activity.

#### Light-dark box

To assess bright-light anxiety, animals were subjected to the light-dark box as previously described by our group^56,57^. A white-black wooden rectangular box (46 × 27 × 30 cm) was divided into two compartments, light (27 × 27 cm) and dark (18 × 27 cm), by a partition. These compartments were connected to each other by central open door (7.5 × 7.5 cm). The light compartment was open at the top, illuminated by a 100 W bulb located 90 cm above the box. The dark compartment had a removable black lid at the top. Each animal was gently placed in the middle of the light compartment, facing away from the open door and allowed to freely explore both compartments for 5 min. The amount of time spent and numbers of entries into the light compartment, and latency of entry into the light compartment after the first entry into the dark division were recorded as indicators of anxiety-like behavior.

#### Elevated plus maze

The elevated plus maze was used to evaluate anxiety-related symptoms in mice as previously described by our group^58,59^. This apparatus was constructed from grey wood with a height of 50 cm. This plus-shaped apparatus consisted of a central area (5 cm × 5 cm), two open arms (30 cm × 5 cm), and two closed arms (30 cm × 5 cm × 15 cm) with an open roof. The animal was gently placed at the middle of the plus maze and allowed to explore freely for 5 min. To increase animal exploratory behavior before anxiety test, mice were subjected to a relatively dark box. The following parameters were measured: (a) time spent in the open arms, (b) time spent in the closed arms, (c) number of entries into the open arms, and (d) number of entries into the closed arms. An entry was considered when all four paws of the animal were into a arm. For the behavioral analysis, percentage of open-arm time (time in open arm/time in open + closed arm × 100), and percentage of open arm entries (number of open arm entries/number of open arm + closed arm entries × 100) were used as indicators of anxiety-like symptoms. The total number of entries into the open and closed arms was used as an indicator of locomotor activity.

### Cytokines measurement in the hippocampus

Two days after the Y-maze test, mice were deeply anaesthetized by an i.p. injection of Ketamine hydrochloride (50 mg/kg; Alfasan) plus Xylazine (5 mg/kg; Alfasan). Animals were perfused with ice-cold pyrogen-free saline and then brain was rapidly removed, placed on an ice-cold surface in a Petri dish filled with saline, and the hippocampus was dissected. The tissues were snap frozen in liquid nitrogen, placed into microcentrifuge tubes and stored at −80 °C until processing. Hippocampal tissues were homogenized in 500 μl buffer (TBS plus 0.2% Triton X-100, 2 mM EDTA, PBS 1 mM PMSF, and protease inhibitor cocktail) and centrifuged at 15,000 g for 15 min at 4 °C. The supernatants were collected and total protein was determined by Micro BCA Protein Assay Kit. IL-1β and BDNF levels were determined using ELISA kits according to kit instructions. The concentration of cytokine protein is presented as pg per mg protein.

### Corticosterone

After the elevated plus maze test, mice were subdivided into two groups: one group was used to measure baseline serum corticosterone levels 2 days after the elevated plus maze test. The other group was used to measure stress-induced plasma corticosterone levels and tested 20 min after the elevated plus maze test. The cardiac puncture was used to collect the blood of mice. Animals were anesthetized by an i.p. injection of Ketamine hydrochloride (50 mg/kg; Alfasan) plus Xylazine (4 mg/kg; Alfasan). The entire sampling procedure was completed within 8 min after removing the animal from its living cage. This is rapid enough to perform a reliable assessment of baseline and stress-induced serum corticosterone levels at the time of sampling, without any undesirable effect of disturbance or anesthesia^44^. The blood was collected into sterile tubes and allowed to clot on ice for a minimum of 15–20 min before centrifugation at 3000 rpm for 10 min. Then, serum was collected into sterile vials and stored at −20 °C until assayed. The specific quantitative sandwich ELISA kit (Bio-Medical Assay Company, Sensitivity: <3 ng/ml) was used to meaure corticosterone according to the manufacturer’s instruction. All samples and standards were assayed in triplicate.

### Data analysis

All data were analyzed using the statistical package of SPSS (IBM). The body weight data were analyzed by three-way repeated measures ANOVA with strain and litter size as between subject factors and age as a within-subject (repeated measure) factor. The other data were analyzed using two-way ANOVA with strain and litter size as between-subject factors. When there was a significant interaction, the data were analyzed by using one-way ANOVA for pairwise comparisons. Pearson correlations were also performed to identify significant relationship between behavioral tests and biochemical parameters. All data are presented as the mean ± standard error of the mean (S.E.M). A *P*-value less than 0.05 was considered statistically significant. A summary of the statistics is shown in Tables S1–8, Supplementary information.

### Data availability statement

The authors confirm that the data will be available upon request.

## Electronic supplementary material

Supplementary information
