# Supplementary material for: Small litter size impairs spatial memory and increases anxiety- like behavior in a strain-dependent manner in male mice
Source: Sci Rep. 2018 Jul 26;8:11281. doi: 10.1038/s41598-018-29595-0 (PMC6062575; doi:10.1038/s41598-018-29595-0)
Supplement: Supplementary file 1 — Supplementary information [file 41598_2018_29595_MOESM1_ESM.docx]

**Supplementary information for:**

Small litter size impairs spatial memory and increases anxiety- like behavior in a strain-dependent manner in male mice

Ali-Akbar Salari ^a,b^*, Hanieh Samadi ^b^, Judith R. Homberg ^c^, Morteza Kosari-Nasab ^a^

^a^ Drug Applied Research Center, Tabriz University of Medical Sciences, Tabriz, Iran

^b^ Salari Institute of Cognitive and Behavioral Disorders (SICBD), Alborz, Karaj, Iran

^c^ Department of Cognitive Neuroscience, Centre for Neuroscience, Donders Institute for Brain, Cognition, and Behaviour, Radboud University Nijmegen Medical Centre, Nijmegen, The Netherlands

***Corresponding author:**

Ali-Akbar Salari;

Drug Applied Research Center,

Tabriz University of Medical Sciences,

P.O. Box 51656-65811, Tabriz, Iran.

Tel./fax: +98 411 3368208.

E-mail: [aa.salari@yahoo.com](mailto:aa.salari@yahoo.com)

**Supplementary Results and Figures**

**Morris Water Maze: Escape latency for each trial of the first day**

As illustrated in Figure S1, repeated measures ANOVA revealed significant trials x litter size (F_(2.43, 107.08)_ =6.35, p = 0.001), and trials x strain x litter size (F_(2.43, 107.08)_ = 7.3, p < 0.001) interactions, however, no significant trials x strain (F_(2.43, 107.08)_ =2.67, p = 0.063) interaction was found. In addition, a between-subject analysis elucidated that there is a significant strain x litter size (F_(1, 44)_ = 4.56, p = 0.038) interaction, although there were no significant main effects for strain (F_(1, 44)_ = 0.93, NS) and litter size (F_(1, 44)_ = 2.75, NS). Subsequent analyses showed that there were no litter size differences for C57BL6 mice (F > 0.69, NS), while NMRI SL mice took significantly longer time to find the platform at trials 3 (F_(1,22)_ = 11.81, p = 0.002) and 4 (F_(1,22)_ = 21.11, p < 0.001) of testing. Significant differences were also found between NMRI SL and C57BL6 SL mice at trials 3 (F_(1,22)_ = 9.86, p = 0.005) and 4 (F_(1,22)_ = 15.63, p = 0.001), in such a way that the NMRI SL mice needed more time to find the platform.

**
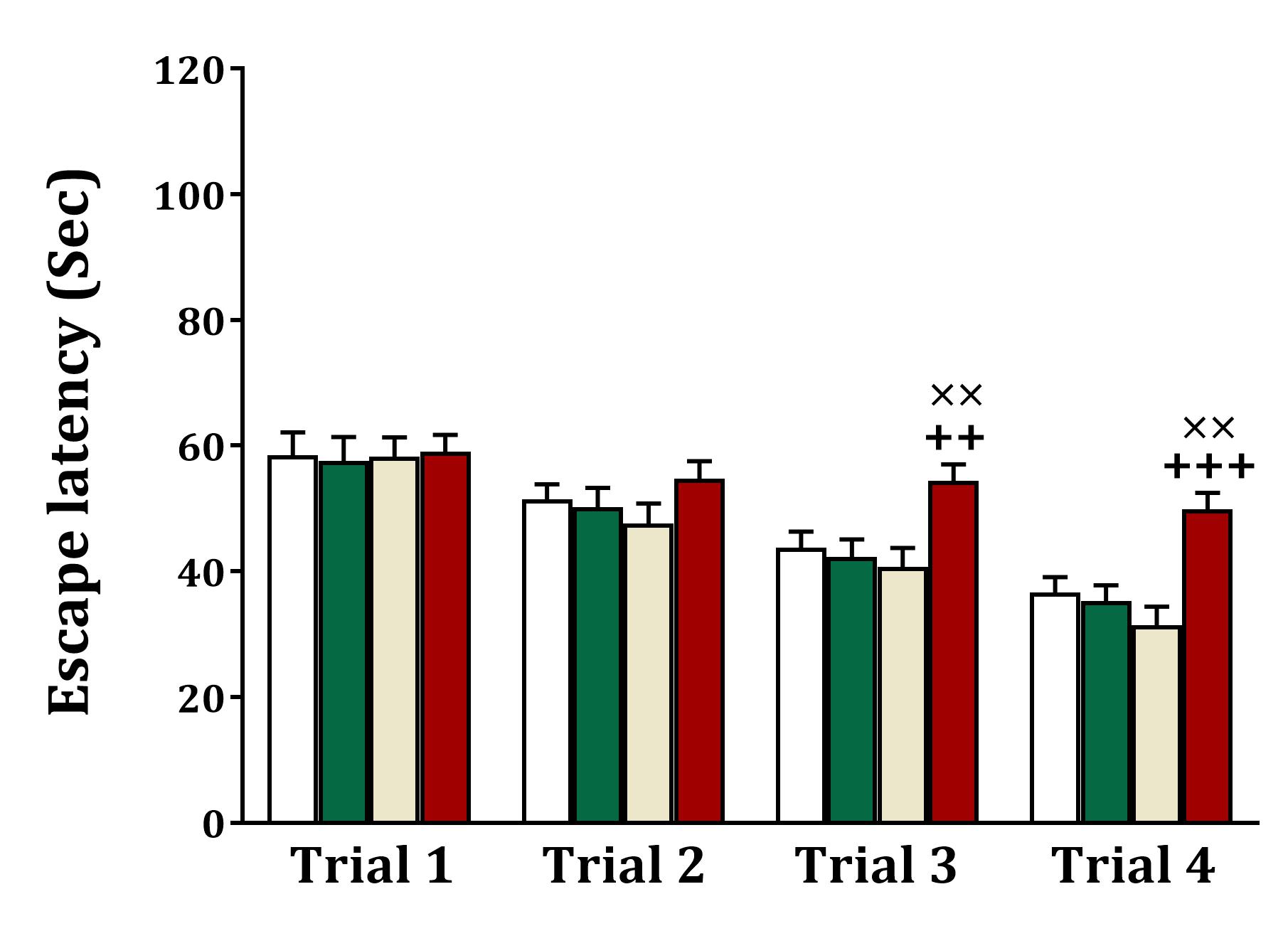
**

**Figure S1.** Effects of neonatal overfeeding in C57BL/6 and NMRI mice on escape latency for each trial of the first day in the Morris water maze. Values are presented as mean + S.E.M.(N=12) of escape latency (Sec). Significant differences following one-way ANOVA: ++*P*<0.01 and +++*P*<0.001, compared to NMRI-CL mice; ××*P*<0.01, compared to C57BL/6-SL mice.

**Locomotor activity**

As shown in Figure S2, there were no significant interactions (F_(1, 44)_ = 0.08, NS; F_(1, 44)_ = 0.13, NS) and main effects of strain (F_(1, 44)_ = 0.45, NS; F_(1, 44)_ = 0.75, NS) and litter size (F_(1, 44)_ = 1.59, NS; F_(1, 44)_ = 1.48, NS) for both total line crossings and rearing, respectively, in the open field test. No significant change was found in the locomotor activity of mice.

**
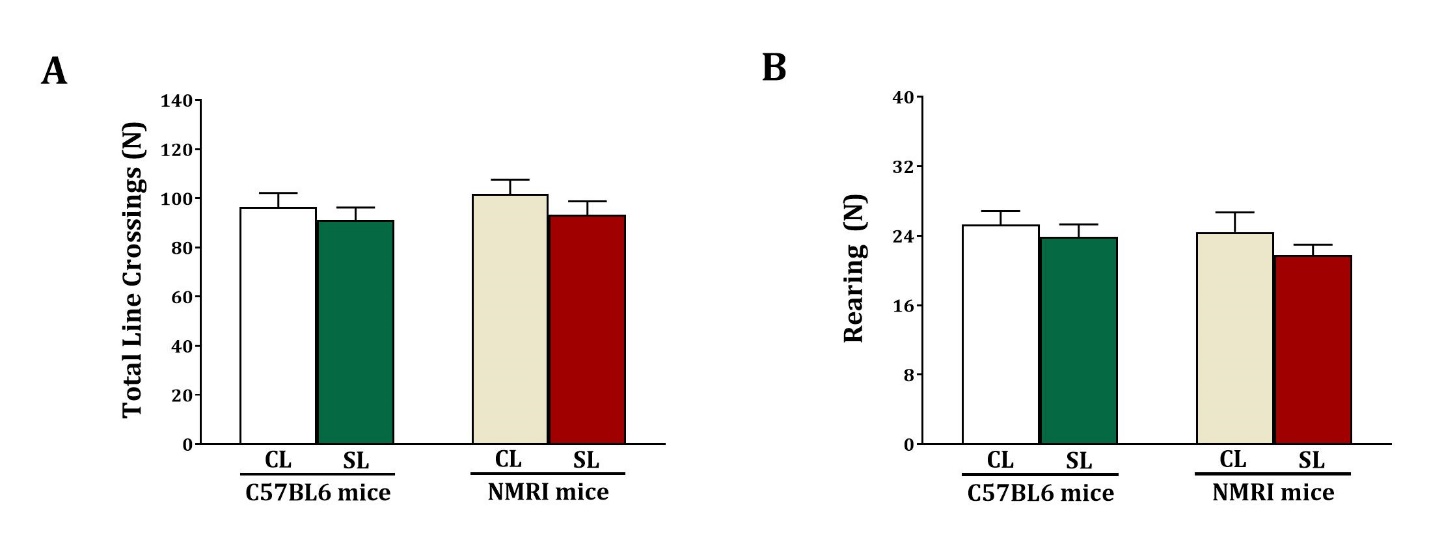
**

**Figure S2.** Effects of neonatal overfeeding in C57BL/6 and NMRI mice on locomotor activity in the open field test. Data are expressed as mean + S.E.M. (N=12).

**
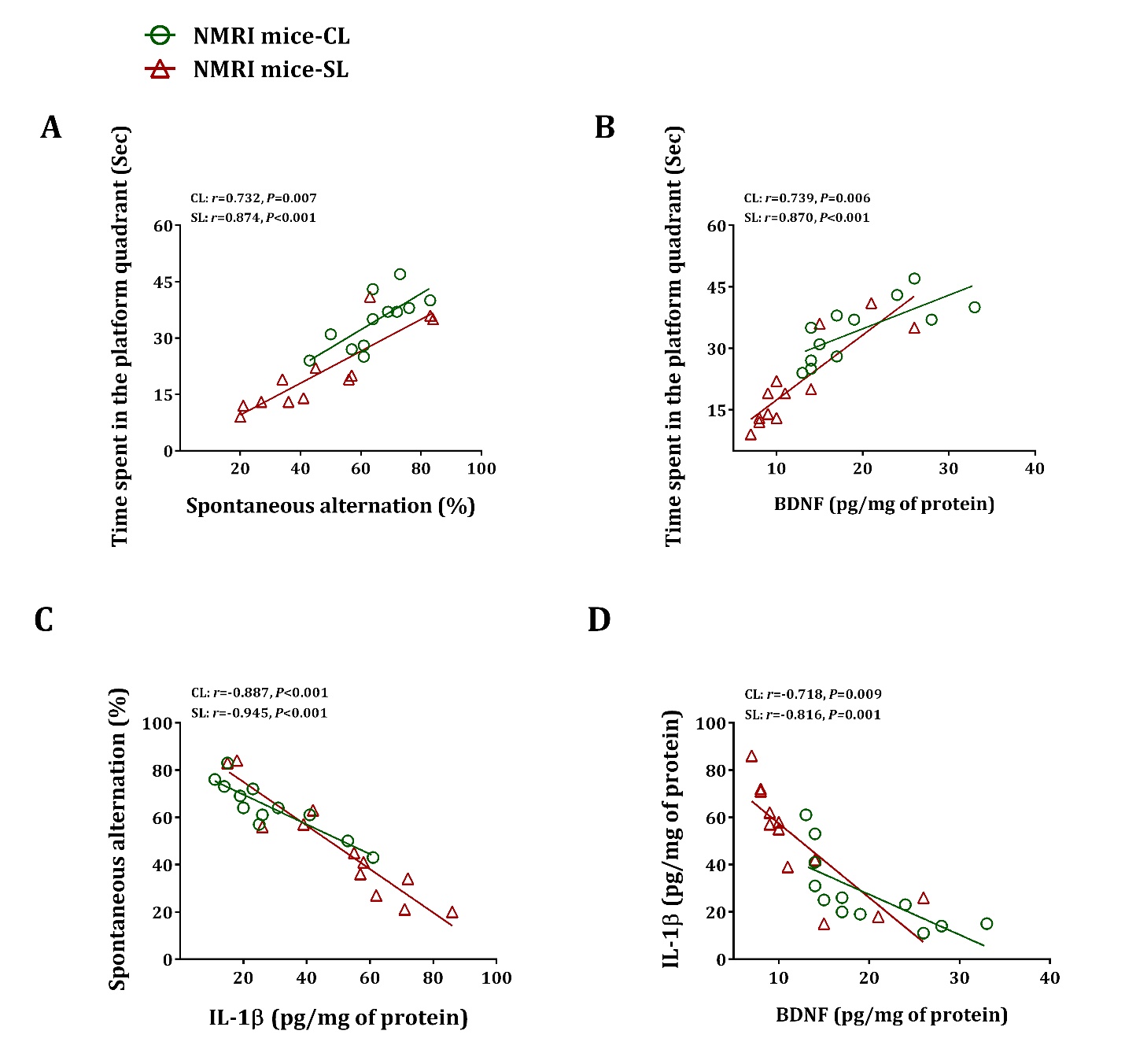
**

**Figure S3.** Pearson correlations between Morris water maze (time spent in the platform quadrant) and Y maze performance (spontaneous alternation %) (A); between Morris water maze performance (time spent in the platform quadrant) and hippocampal BDNF levels (B); between Y maze performance (spontaneous alternation %) and hippocampal IL-1β levels (C) and between IL-1β and BDNF levels in the hippocampus (D).

**
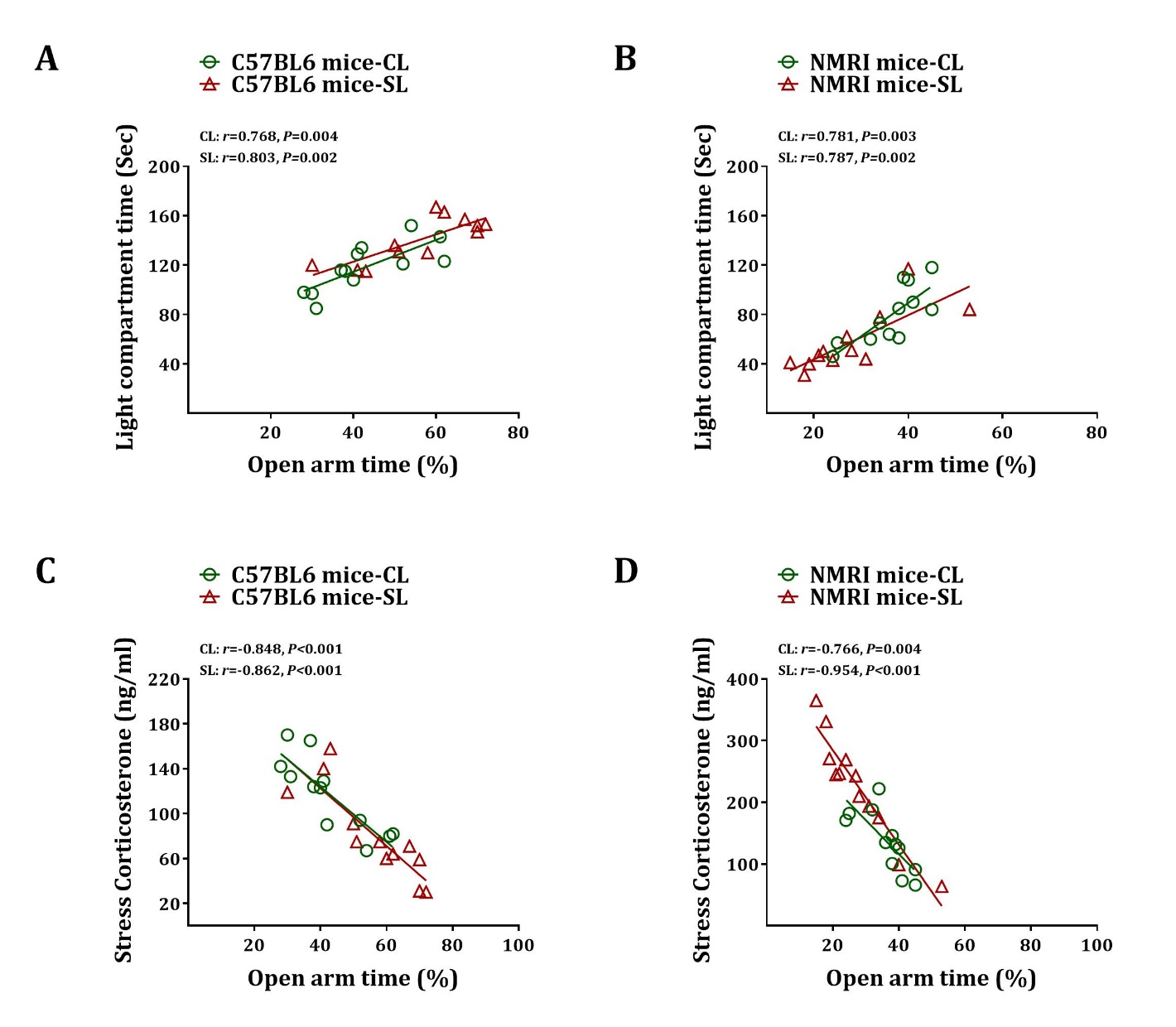
Figure S4.** Pearson correlations between light-dark box (light compartment time) and elevated plus maze test performance (open arm time %) (A: C57BL/6 mice and B: NMRI mice) and between elevated plus maze test performance (open arm time %) and stress-induced corticosterone levels (C: C57BL/6 mice and D: NMRI mice).

**Summary Statistics**

| **Body weight** | | | | | |
| --- | --- | --- | --- | --- | --- |
| **Days** | **Statistics**  **Groups** | **N**  **Statistics** | **Mean**  **Statistics** | **Mean**  **Std. Error** | **Std. Deviation**  **Statistics** |
| **Day 3** | C57BL6 mice-CL | 12 | 1.525 | 0.0719 | 0.2491 |
|  | C57BL6 mice-SL | 12 | 1.508 | 0.0609 | 0.2109 |
|  | NMRI mice-CL | 12 | 1.558 | 0.0499 | 0.1730 |
|  | NMRI mice-SL | 12 | 1.508 | 0.0514 | 0.1782 |
| **Day 10** | C57BL6 mice-CL | 12 | 3.817 | 0.3402 | 1.1785 |
|  | C57BL6 mice-SL | 12 | 6.367 | 0.4614 | 1.5985 |
|  | NMRI mice-CL | 12 | 4.783 | 0.4409 | 1.5272 |
|  | NMRI mice-SL | 12 | 8.183 | 0.5381 | 1.8639 |
| **Day 20** | C57BL6 mice-CL | 12 | 8.358 | 0.4996 | 1.7307 |
|  | C57BL6 mice-SL | 12 | 10.050 | 0.9111 | 3.1561 |
|  | NMRI mice-CL | 12 | 10.725 | 0.6128 | 2.1227 |
|  | NMRI mice-SL | 12 | 13.542 | 0.9462 | 3.2776 |
| **Day 40** | C57BL6 mice-CL | 12 | 17.467 | 0.7354 | 2.5475 |
|  | C57BL6 mice-SL | 12 | 20.425 | 1.2729 | 4.4095 |
|  | NMRI mice-CL | 12 | 22.558 | 1.3373 | 4.6324 |
|  | NMRI mice-SL | 12 | 28.433 | 1.6369 | 5.6704 |
| **Day 80** | C57BL6 mice-CL | 12 | 24.683 | 0.9872 | 3.4197 |
|  | C57BL6 mice-SL | 12 | 29.108 | 1.7958 | 6.2207 |
|  | NMRI mice-CL | 12 | 30.050 | 1.4469 | 5.0123 |
|  | NMRI mice-SL | 12 | 37.442 | 1.6359 | 5.6670 |

**Table S1.** Summary Statistics for the body weight data.

| **Open Field; Locomotor Activity** | | | | | |
| --- | --- | --- | --- | --- | --- |
| **Parameter** | **Statistics**  **Groups** | **N**  **Statistics** | **Mean**  **Statistics** | **Mean**  **Std. Error** | **Std. Deviation**  **Statistics** |
| **Total line crossings** | C57BL6 mice-CL | 12 | 96.50 | 5.667 | 19.631 |
|  | C57BL6 mice-SL | 12 | 91.17 | 5.081 | 17.601 |
|  | NMRI mice-CL | 12 | 101.83 | 5.711 | 19.784 |
|  | NMRI mice-SL | 12 | 93.25 | 5.545 | 19.208 |
| **Rearing** | C57BL6 mice-CL | 12 | 25.25 | 1.582 | 5.479 |
|  | C57BL6 mice-SL | 12 | 23.83 | 1.461 | 5.060 |
|  | NMRI mice-CL | 12 | 24.42 | 2.261 | 7.833 |
|  | NMRI mice-SL | 12 | 21.75 | 1.207 | 4.181 |

**Table S2.** Summary Statistics for the locomotion data.

| **Morris Water Maze** | | | | | |
| --- | --- | --- | --- | --- | --- |
| **Parameter** | **Statistics**  **Groups** | **N**  **Statistics** | **Mean**  **Statistics** | **Mean**  **Std. Error** | **Std. Deviation**  **Statistics** |
| **Escape latency for trial 1 of the first day** | C57BL6 mice-CL | 12 | 58.42 | 3.677 | 12.738 |
|  | C57BL6 mice-SL | 12 | 57.58 | 3.825 | 13.249 |
|  | NMRI mice-CL | 12 | 58.25 | 3.073 | 10.644 |
|  | NMRI mice-SL | 12 | 59.00 | 2.733 | 9.468 |
| **Escape latency for trial 2 of the first day** | C57BL6 mice-CL | 12 | 51.50 | 2.366 | 8.196 |
|  | C57BL6 mice-SL | 12 | 50.25 | 3.028 | 10.489 |
|  | NMRI mice-CL | 12 | 47.58 | 3.216 | 11.139 |
|  | NMRI mice-SL | 12 | 54.75 | 2.777 | 9.621 |
| **Escape latency for trial 3 of the first day** | C57BL6 mice-CL | 12 | 43.75 | 2.614 | 9.057 |
|  | C57BL6 mice-SL | 12 | 42.25 | 2.858 | 9.901 |
|  | NMRI mice-CL | 12 | 40.75 | 2.995 | 10.376 |
|  | NMRI mice-SL | 12 | 54.42 | 2.615 | 9.060 |
| **Escape latency for trial 4 of the first day** | C57BL6 mice-CL | 12 | 36.67 | 2.429 | 8.414 |
|  | C57BL6 mice-SL | 12 | 35.25 | 2.538 | 8.792 |
|  | NMRI mice-CL | 12 | 31.42 | 2.983 | 10.335 |
|  | NMRI mice-SL | 12 | 49.83 | 2.677 | 9.272 |
| **Escape latency**  **(Day 1)** | C57BL6 mice-CL | 12 | 47.58 | 2.491 | 8.628 |
|  | C57BL6 mice-SL | 12 | 46.33 | 2.726 | 9.442 |
|  | NMRI mice-CL | 12 | 44.50 | 2.922 | 10.122 |
|  | NMRI mice-SL | 12 | 54.58 | 2.372 | 8.218 |
| **Escape latency**  **(Day 2)** | C57BL6 mice-CL | 12 | 38.42 | 2.835 | 9.821 |
|  | C57BL6 mice-SL | 12 | 37.25 | 2.129 | 7.375 |
|  | NMRI mice-CL | 12 | 34.67 | 1.994 | 6.906 |
|  | NMRI mice-SL | 12 | 44.75 | 2.568 | 8.895 |
| **Escape latency**  **(Day 3)** | C57BL6 mice-CL | 12 | 27.75 | 1.508 | 5.225 |
|  | C57BL6 mice-SL | 12 | 27.92 | 1.893 | 6.557 |
|  | NMRI mice-CL | 12 | 25.58 | 1.712 | 5.931 |
|  | NMRI mice-SL | 12 | 34.00 | 2.874 | 9.954 |
| **Escape latency**  **(Day 4)** | C57BL6 mice-CL | 12 | 21.33 | 1.479 | 5.123 |
|  | C57BL6 mice-SL | 12 | 22.17 | 1.918 | 6.645 |
|  | NMRI mice-CL | 12 | 18.42 | 1.540 | 5.334 |
|  | NMRI mice-SL | 12 | 25.17 | 1.961 | 6.793 |
| **Escape latency**  **(Day 5)** | C57BL6 mice-CL | 12 | 14.50 | 0.981 | 3.398 |
|  | C57BL6 mice-SL | 12 | 13.67 | 1.082 | 3.750 |
|  | NMRI mice-CL | 12 | 11.92 | 0.839 | 2.906 |
|  | NMRI mice-SL | 12 | 17.50 | 1.844 | 6.389 |
| **Time spent in the platform quadrant** | C57BL6 mice-CL | 12 | 30.25 | 2.175 | 7.533 |
|  | C57BL6 mice-SL | 12 | 32.00 | 1.826 | 6.325 |
|  | NMRI mice-CL | 12 | 34.33 | 2.123 | 7.353 |
|  | NMRI mice-SL | 12 | 21.17 | 3.037 | 10.521 |
| **Platform crossings** | C57BL6 mice-CL | 12 | 5.25 | 0.329 | 1.138 |
|  | C57BL6 mice-SL | 12 | 5.33 | 0.449 | 1.557 |
|  | NMRI mice-CL | 12 | 6.17 | 0.490 | 1.697 |
|  | NMRI mice-SL | 12 | 4.08 | 0.583 | 2.021 |

**Table S3.** Summary Statistics for the Morris water maze data.

| **Y Maze** | | | | | |
| --- | --- | --- | --- | --- | --- |
| **Parameter** | **Statistics**  **Groups** | **N**  **Statistics** | **Mean**  **Statistics** | **Mean**  **Std. Error** | **Std. Deviation**  **Statistics** |
| **Spontaneous**  **alternation** | C57BL6 mice-CL | 12 | 72.33 | 3.791 | 13.131 |
|  | C57BL6 mice-SL | 12 | 69.33 | 3.920 | 13.580 |
|  | NMRI mice-CL | 12 | 64.42 | 3.230 | 11.188 |
|  | NMRI mice-SL | 12 | 47.25 | 6.298 | 21.818 |
| **Total arm entries** | C57BL6 mice-CL | 12 | 31.08 | 1.583 | 5.485 |
|  | C57BL6 mice-SL | 12 | 28.75 | 1.962 | 6.797 |
|  | NMRI mice-CL | 12 | 25.33 | 1.509 | 5.228 |
|  | NMRI mice-SL | 12 | 23.83 | 1.637 | 5.670 |

**Table S4.** Summary Statistics for the Y maze data.

| **Hippocampal IL-1β and BDNF** | | | | | |
| --- | --- | --- | --- | --- | --- |
| **Parameter** | **Statistics**  **Groups** | **N**  **Statistics** | **Mean**  **Statistics** | **Mean**  **Std. Error** | **Std. Deviation**  **Statistics** |
| **IL-1β** | C57BL6 mice-CL | 12 | 24.50 | 2.776 | 9.615 |
|  | C57BL6 mice-SL | 12 | 25.42 | 2.840 | 9.839 |
|  | NMRI mice-CL | 12 | 28.25 | 4.546 | 15.749 |
|  | NMRI mice-SL | 12 | 50.08 | 6.463 | 22.387 |
| **BDNF** | C57BL6 mice-CL | 12 | 21.17 | 1.984 | 6.873 |
|  | C57BL6 mice-SL | 12 | 22.25 | 2.339 | 8.103 |
|  | NMRI mice-CL | 12 | 19.50 | 1.913 | 6.626 |
|  | NMRI mice-SL | 12 | 12.33 | 1.680 | 5.821 |

**Table S5.** Summary Statistics for the hippocampal IL-1β and BDNF data.

| **Light-Dark Box** | | | | | |
| --- | --- | --- | --- | --- | --- |
| **Parameter** | **Statistics**  **Groups** | **N**  **Statistics** | **Mean**  **Statistics** | **Mean**  **Std. Error** | **Std. Deviation**  **Statistics** |
| **Light compartment time** | C57BL6 mice-CL | 12 | 118.42 | 5.663 | 19.617 |
|  | C57BL6 mice-SL | 12 | 140.58 | 5.296 | 18.347 |
|  | NMRI mice-CL | 12 | 79.67 | 6.752 | 23.391 |
|  | NMRI mice-SL | 12 | 57.25 | 7.081 | 24.529 |
| **Light compartment entries** | C57BL6 mice-CL | 12 | 17.50 | 1.190 | 4.123 |
|  | C57BL6 mice-SL | 12 | 22.17 | 1.186 | 4.108 |
|  | NMRI mice-CL | 12 | 11.42 | 1.062 | 3.679 |
|  | NMRI mice-SL | 12 | 8.25 | 0.605 | 2.094 |
| **Latency of entry into the light** | C57BL6 mice-CL | 12 | 6.75 | 0.708 | 2.454 |
|  | C57BL6 mice-SL | 12 | 5.42 | 0.583 | 2.021 |
|  | NMRI mice-CL | 12 | 9.17 | 0.683 | 2.368 |
|  | NMRI mice-SL | 12 | 14.58 | 1.621 | 5.616 |

**Table S6.** Summary Statistics for the light-dark box data.

| **Elevated Plus Maze** | | | | | |
| --- | --- | --- | --- | --- | --- |
| **Parameter** | **Statistics**  **Groups** | **N**  **Statistics** | **Mean**  **Statistics** | **Mean**  **Std. Error** | **Std. Deviation**  **Statistics** |
| **Open arm time (%)** | C57BL6 mice-CL | 12 | 43.00 | 3.371 | 11.677 |
|  | C57BL6 mice-SL | 12 | 56.17 | 3.849 | 13.334 |
|  | NMRI mice-CL | 12 | 36.42 | 1.952 | 6.762 |
|  | NMRI mice-SL | 12 | 27.67 | 3.095 | 10.722 |
| **Open arm entries (%)** | C57BL6 mice-CL | 12 | 22.50 | 1.505 | 5.214 |
|  | C57BL6 mice-SL | 12 | 27.25 | 1.601 | 5.545 |
|  | NMRI mice-CL | 12 | 17.75 | 0.962 | 3.334 |
|  | NMRI mice-SL | 12 | 13.00 | 0.921 | 3.191 |
| **Total arm entries** | C57BL6 mice-CL | 12 | 26.25 | 1.543 | 5.345 |
|  | C57BL6 mice-SL | 12 | 28.00 | 2.071 | 7.173 |
|  | NMRI mice-CL | 12 | 21.00 | 1.237 | 4.285 |
|  | NMRI mice-SL | 12 | 22.50 | 1.968 | 6.816 |

**Table S7.** Summary Statistics for the elevated plus maze data.

| **Corticosterone** | | | | | |
| --- | --- | --- | --- | --- | --- |
| **Parameter** | **Statistics**  **Groups** | **N**  **Statistics** | **Mean**  **Statistics** | **Mean**  **Std. Error** | **Std. Deviation**  **Statistics** |
| **Baseline**  **corticosterone** | C57BL6 mice-CL | 12 | 26.33 | 2.916 | 10.103 |
|  | C57BL6 mice-SL | 12 | 24.67 | 2.391 | 8.283 |
|  | NMRI mice-CL | 12 | 29.75 | 3.184 | 11.030 |
|  | NMRI mice-SL | 12 | 33.17 | 4.034 | 13.973 |
| **Stress**  **corticosterone** | C57BL6 mice-CL | 12 | 116.58 | 9.760 | 33.808 |
|  | C57BL6 mice-SL | 12 | 81.08 | 11.495 | 39.819 |
|  | NMRI mice-CL | 12 | 136.25 | 13.856 | 48.000 |
|  | NMRI mice-SL | 12 | 226.08 | 24.858 | 86.110 |

**Table S8.** Summary Statistics for the corticosterone data.
